# Student perception of the effect of problem familiarity on group discussion quality in a problem-based learning environment

**DOI:** 10.3205/zma001237

**Published:** 2019-05-16

**Authors:** Mohamed Elhassan Abdalla, Mohamed Ahmed Eladl

**Affiliations:** 1 University of Sharjah, College of Medicine, Medical Education Center, Sharjah, United Arab Emirates; 2 University of Sharjah, College of Medicine, Basic Medical Sciences, Sharjah, United Arab Emirates

**Keywords:** Problem-based learning, scenarios, familiarity, tutorial discussion

## Abstract

**Introduction: **Problem-based learning (PBL) is a student-centred approach to learning using health problem scenarios to trigger the learning process. Several factors contribute to the role of the problem scenarios in stimulating student learning. One of those factors is the student’s familiarity and knowledge about the problem itself. This may affect the challenge and stimulate the student discussion in the tutorial group. No previous research studied the impact of reusing the case scenarios on the group discussion. This study explored the effect of student familiarity of the problems as a result of reusing the case scenarios on the discussion quality in the tutorial session.

**Methods: **A qualitative study was used primarily to explore an understanding of the underlying opinions of the medical students of first and second academic year in the college of Medicine, University of Sharjah, UAE. Direct-discussion groups were arranged, and an open-ended online questionnaire was provided.

**Results: **The results of the study showed that fore-knowledge about the case scenario had no significant adverse effect on the discussion. Students stated that the facilitators played a vital role in maintaining the excellent quality of the discussion.

**Discussion:** Reuse of problem scenarios in PBL does not hurt the quality of the discussion, provided that the group dynamics are maintained.

## Zusammenfassung

**Einführung: **Problembasiertes Lernen (PBL) ist ein auf Studenten bezogener Ansatz, um zu lernen, Gesundheitsproblem-Szenarien für das Auslösen eines Lerneffekts zu nutzen. Mehrere Faktoren tragen zur Rolle von Problemszenarien bei, um Studenten zum Lernen anzuregen. Einer der Faktoren ist die Vertrautheit von Studenten mit einem Problem bzw. deren Kenntnis des Problems selbst. Dies kann einen Einfluss auf die Herausforderung und Stimulierung der Studentendiskussion in einer Lerngruppe haben. Bisherige Forschungen haben nicht den Einfluss auf Gruppendiskussionen untersucht, wenn Fallstudien-Szenarien erneut verwendet werden. Die vorliegende Studie hat die Auswirkungen untersucht, die eine Vertrautheit der Studenten mit den Problemen infolge einer erneuten Verwendung von Fallstudien-Szenarien auf die Diskussionsqualität im Tutoriumskurs hat.

**Methoden: **Eine qualitative Studie wurde vorrangig genutzt, um das Verständnis zugrunde liegender Meinungen von Medizinstudenten des ersten und zweiten akademischen Jahres am College of Medicine, University of Sharjah, VAE, zu untersuchen. Direkte Diskussionsgruppen wurden zusammengestellt und es wurde ein offen endender Online-Fragebogen zur Verfügung gestellt.

**Ergebnisse: **Die Ergebnisse der Studie zeigten, dass Vorkenntnisse von einem Fallstudien-Szenario keine signifikanten nachteiligen Auswirkungen auf die Diskussion hatten. Studenten gaben an, dass die Moderatoren eine wesentliche Rolle dabei spielten, die hohe Qualität der Diskussion beizubehalten.

**Diskussion: **Die erneute Verwendung von Problemszenarien im PBL beeinträchtigt nicht die Qualität der Diskussion, vorausgesetzt, die Gruppendynamik bleibt erhalten.

## Introduction

Problem-based learning (PBL) is a learning approach started in the 1960s at McMaster University, Canada. It is a student-centered approach that triggers learning by having a problem scenario presented early in the learning process [1], [2], [3], [4], [5], [6]. Each group of students addresses the problem in the presence of a facilitator, following the process of the seven jump [7], [8]. 

The role of the problem scenario is to encourage students to activate their prior knowledge and to stimulate students’ interest in the subject matter [9], and hence to engage the students in an active discussion and to create a positive learning environment [10], [11], [12]. Several factors affect the quality of the PBL scenario and its effectiveness in stimulating discussions. These factors include the reality of the scenarios, the variety of experiences, the degree of challenge, supporting group work and the ability to activate prior knowledge [13], [14], [15], [16]. PBL aims at helping students to define new learning needs.

Familiarity with the problems and how it affects the degree of challenge and the quality of students’ discussion in the tutorial group has been studied by various authors. Some studied this with a primary focus on the effect of the discussion regarding long-term retention of knowledge. However, no previous research studied the impact of the immediate recognition of a case scenario resulting from its reuse on the group discussion. This gap in the research indicates a need for further study. 

This study was conducted at the College of Medicine at the University of Sharjah, where PBL is used as a method of teaching and learning, and as a trigger for the discussion of the curriculum themes. The PBL case scenarios are reused for many years. During the evaluation of the curriculum implementation, the college educational leaders were investigating the effect of reusing the case scenarios with regard to the quality of student discussions. The study was conducted with seeking the permission of the college administration as part of an evaluation process of the PBL by students. The process initially depended on the feedback collected from students through questionnaires after each problem discussion. The questionnaires did not contain questions about the effect of being familiar with the case scenarios beforehand. 

The objective of this study was to explore the students’ opinion how being familiar with the scenarios as a result of reusing them impacted on the quality of the discussion during the PBL tutorial session. 

## Methods

This was a qualitative study conducted with the participation of students in years one and two in the College of Medicine, University of Sharjah. Data were collected through direct discussion within groups and from an online questionnaire with open ended questions. For the discussion, students were placed in the same groups they had originally been assigned to for the problem-based learning. Students were made aware of the research through a formal notification process in the college and were informed that participation was voluntary. A total of 20 group discussions were conducted, ten for each academic year, with 7–10 participants in each group. 

The PBL facilitators conducted the discussions after being trained by the researchers. The duration of each discussion was between 30 and 45 minutes. The discussions were carried out at the same time and on the same day for each academic year, after the end of the scheduled PBL tutorial session, to maximise the attendance of students. Students were given the option to leave if they did not want to participate or if they had other commitments (as the discussions were conducted towards the end of the working day).

The discussion addressed two points:

1. For approximately how many “problems” did students attempt to know the scenario and learning objectives from previous years’ colleagues in advance; and
2. If they knew the learning objectives of the problem from other students, did this affect their discussion in the tutorial groups? If so, how?

In the discussion, the facilitator asked a question, allowed 3-5 minutes for the participants to think and then asked them to respond. Responses to both questions were collected in such a way that every one of the students provided an answer. Then, an open free discussion among the group members was allowed and the facilitator worked to monitor the group dynamics. Notes on the discussion were taken by the facilitator, as there were only two points of discussion. The discussion responses were summarised by each group facilitator and provided to the researchers. 

To increase the number of responses, the two points discussed were also sent to students in the form of an online questionnaire, using SurveyMonkey®, for those who could not attend the discussions. 48 opened the questionnaire, but only 30 provided responses.

All responses from the group discussions and the online questionnaires were read and analysed by the researchers, using the content analysis technique to generate common themes. 

## Results

Almost all students in all the groups and those who respond to the questionnaire had some knowledge beforehand about some or all of the problem scenarios they were going to address in the tutorial session. The title or the summary of the scenario was the most common information they knew. A few students tended to identify the learning objectives of particular problems or asked about specific points to discuss. The source of their information were usually senior students. Below are some of the students’ responses.

- “We have a rough idea about at least the main problems in the scenario almost every week.”
- “I knew the problem only, not the objectives.”
- “Mostly from my colleagues, who had knowledge from senior students…”
- “In year-one scenarios, I had no idea about the objectives before the session; then although I had files from my senior friends, I did not open the objectives.”

Most students thought that their prior knowledge about the problem had a positive impact on the quality and quantity of the discussion they were to have. They stated that, by having this knowledge beforehand, they have more information to share during the discussion and they had more contributions and involvement.

One point the students mentioned was that, through knowledge about the problem scenarios, they could pinpoint the critical points to be discussed, and this would facilitate the production of the learning outcomes. Examples of students’ responses are below.

- “It will enhance my discussion…I would at least have a superficial idea of what is going on, to better-guided brainstorm.”
- “It does not decrease the discussion, but it sometimes stimulates some students to read about the problem before coming to the PBL session, which can affect the session negatively.”
- “I do not think it would affect us in that way; it might even help with more discussion since some members of the group might have already prepared beforehand…”
- “It leads to more discussion; I will prepare myself.”

A few students believed that knowing the learning objectives of the problem from other students affects the discussion negatively, as it limits thinking and generation of probabilities and leads to the dominance of those who know over those who do not know. Below are some of the students’ responses.

- “Yes, it will affect my discussion. I think it will be more passive because I already know the objectives.”
- “It leads to less discussion.”
- “Yes, because then I will not think about all the possibilities…”
- “It is very annoying because the discussion is only limited to one or two students who dominate the discussion.”
- “It will affect it negatively, so some students will jump by introducing the objectives rather than think about the finding.”
- “Considering the fact that there are a few students who know the learning objectives of the scenario in advance, it affects students who are not aware of the objectives, and this leads to less discussion within the group.”

Very few students believed that there was absolutely no effect on the discussion when they were not aware of the learning objectives of the problem.

- “It does not affect the discussion much since we know what the problem is about, but we don't know anything about the topic.”
- “No, it really has no effect knowing or not knowing the problem.”

Students in all the groups agreed that the quality of the discussion depended on the manner in which the facilitator carried out his/her role, and that it was not affected by whether they knew about the problem beforehand or not.

- “It is all about the facilitator.”
- “It will not affect the quality of the discussion as long as the facilitator can direct the discussion.”

## Discussion

As per the results obtained, knowledge about the case scenario but not the learning objectives may lead to better discussion as the level of previous knowledge may increase. Sockalingam and Schmidt [17] explored the effect of the cumulative prior knowledge and experience a student may have about the case. They concluded that this prior knowledge and expertise has a positive impact on student learning. Still, they believe that some level of unfamiliarity with the case may be preferable, as it will lead to the generation of more questions and discussion among students. Thus, their conclusion partly supports the results of our study: students still will not know all the aspects of the problem; they know only the summary and some of the points needed to generate learning objectives.

In another study about familiarity (prior knowledge) with the problem, Soppe et al. [9] reported that students perceived the familiar problem as a high-quality one when compared to a non-familiar problem, but there was no difference in the quality of the students’ discussion.

On the other hand, the results of this study indicated that knowledge of the learning objectives enhanced the discusion but had a negative impact on its quality. This is also supported by the conclusions from the studies mentioned above and also by the study by Mauffette et al. [2] who indicated that the discussion relating to the problem is affected by many factors, such as the level of challenges perceived by students and how a student conceptualizes the problem from his/her context. These factors will likely be affected when the students know the learning objectives of the case scenario beforehand.

Students in this study indicated that the quality of the discussion depends greatly on the facilitator. The importance of the facilitator's role is well recognized. Maudsley (1999), reported that the PBL facilitator should be a process expert rather than subject expert [18]. Yee et al. (2006) have also concluded that *“facilitator can make or break the session”*; they argue that facilitators can motivate student learning even if the triggers are not of the most effective quality [19]. The finding from Yee et al. are highly compatible with our findings. We did not come across any article that does not value the role of the facilitator in PBL. 

The source of information for students about the problem scenarios is the senior peers; which is considered a difficult source to control. 

## Conclusion

The results of this study suggest different effects of prior knowledge about the problem scenario on the quality of the discussion within the tutorial group. The effects depend on what is known by students. The results also suggest that reusing problem scenarios in PBL does not hurt the quality of the discussions, provided that the group dynamics are maintained by the facilitator.

## Limitations

The study was somewhat limited in that it depended solely on student perceptions; a more objective observation of the discussion in the tutorial groups may lead to better results. The results may need to be supported by another measurement of the quality of the discussion, such as recordings of some of the tutorial sessions or qualitative data from the group facilitators’ opinions. Further involvement of the facilitators as research participants may add to the results of this study.

## Competing interests

The authors declare that they have no competing interests. 
